# Influence of Pore Size of Mesoporous Silica on Physical Stability of Overloaded Celecoxib Glass

**DOI:** 10.1021/acs.molpharmaceut.4c01482

**Published:** 2025-04-04

**Authors:** Xue Han, Kohsaku Kawakami

**Affiliations:** † Research Center for Macromolecules and Biomaterials, National Institute for Materials Science, 1-1 Namiki, Tsukuba, Ibaraki 305-0044, Japan; ‡ Graduate School of Science and Technology, University of Tsukuba, 1-1-1 Tennodai, Tsukuba, Ibaraki 305-8577, Japan

**Keywords:** glass, mesoporous silica, crystallization, cooperatively rearranging region, broadband dielectric spectroscopy, differential scanning calorimetry

## Abstract

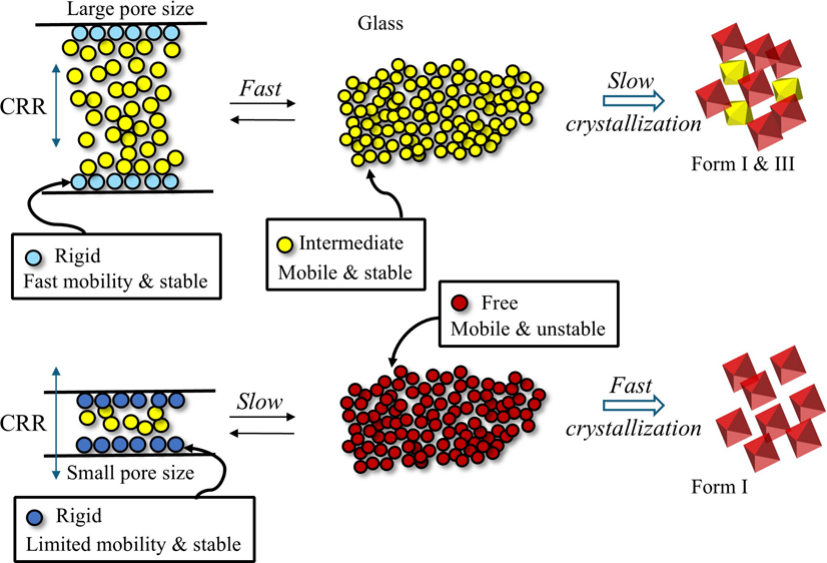

The stabilization mechanism of mesoporous silica (MS) of two different pore sizes (21 and 2.5 nm) on overloaded celecoxib (CEL) glass was investigated. Differential scanning calorimetry (DSC) measurements revealed the presence of three fractions with different molecular mobilities: free, intermediate, and rigid ones. The free fraction exhibited cold crystallization during DSC heating and was assumed to have almost the same properties as those of the bulk molecules. The rigid fraction did not exhibit either glass transition or cold crystallization behavior, which should be stabilized by interactions with the MS surface. The remaining molecules exhibited glass transition behavior without any tendency toward cold crystallization during heating, which is called the intermediate fraction. The molecular dynamics of each fraction was investigated by using broadband dielectric spectroscopy (BDS). While the intermediate and free fractions exhibited comparable mobility, the rigid fraction demonstrated pore-size-dependent behavior: enhanced and suppressed molecular mobility was observed for the rigid fraction confined in 21 and 2.5 nm-pores, respectively. Isothermal crystallization of CEL glass was investigated using DSC and BDS at 95 °C. The results revealed that the CEL glass mixed with MS with large pores exhibited slower crystallization compared to the CEL glass without MS, whereas accelerated crystallization was observed for the CEL mixed with a small amount of MS of small pores. The pore size of 21 nm was much larger than the cooperatively rearranging region (CRR) of the CEL glass, whereas the pore size of 2.5 nm was comparable to that. When the pore size was larger than that of the CRR, most of the loaded CEL molecules behaved as an intermediate fraction, presumably because the molecules could exchange inside and outside the pore. In contrast, the exchange was not likely to proceed when the pore size was comparable to or smaller than that of the CRR, leaving a large free fraction. This finding provides a deep understanding of the stabilization mechanism of overloaded pharmaceutical glass by using mesoporous materials.

## 1 Introduction

Drugs with poor aqueous solubility often face challenges with low oral bioavailability when the solubility or dissolution processes limit absorption. A promising strategy for improving low bioavailability in such cases is transforming the crystalline form of the drug into glass.^1−4^ Because the glass state is thermodynamically unstable, it may crystallize during storage and/or immediately after contact with aqueous media.^5^ An approach to stabilizing drugs involves using porous materials such as mesoporous silica (MS).^6−8^ Its stabilization mechanism is generally assumed to include two factors: the direct interaction of guest molecules with the surface of the material and the confinement effect in small pores.^9^ Hydrogen bonding between silanol groups on the surface and guest molecules is mainly responsible for the former mechanism. The capture of molecules in small pores influences their molecular mobility; this is known as the confinement effect. As molecular mobility is an important factor that affects the crystallization behavior,^10,11^ molecules with limited mobility in the pores should have a lower tendency to crystallize. Moreover, crystals cannot grow in pores unless the space provided is sufficiently large for nucleation and crystal growth.

Understanding the influence of the properties of porous material, including pore size, particle size, and surface chemistry, on the physical stability of guest drugs is of paramount importance. Surface adsorption mechanisms include hydrogen bonding, electrostatic interactions, and hydrophobic interactions. The effectiveness of drug loading and release may be influenced by the adsorption mechanism.^7^ MS with the same pore size but different particle sizes exhibited different stabilization effects for overloaded simvastatin glass.^12^ This can be explained by the more effective inhibition of the crystal growth of bulk glass by small particles and easier exchange of the inside and outside molecules of the MS for the smaller particles. Regarding the pore size effect, when prednisolone glass was entrapped within pores of 1.60 and 2.16 nm, larger pores were reported to have a higher stabilization effect.^13^ In contrast, flufenamic acid glass was found to be stabilized in smaller pores including 3.2 nm pores of MCM and 7.1 nm pores of SBA, whereas crystallization was observed in the 29 nm pores of MCF silica.^14^ Similarly, nifedipine glass entrapped in pores smaller than 12 nm exhibited better stability than that in pores of 50–198 nm,^15^ despite both pore sizes being significantly larger than the molecular size. These apparently contradictory observations indicate that more systematic studies are required to provide general ideas regarding the stabilization mechanism of mesoporous materials. A comparison of the relative pore sizes is not likely to provide much information, but more focus on the relationship between the pore size and glass properties is required. Moreover, the stabilization effect only for the entrapped molecules in the pores is not sufficient for the use of MS as a pharmaceutical excipient to avoid an increase in the formulation volume. The stabilization of overloaded drugs is of practical importance.

Extensive efforts have also been made from a formulation viewpoint to demonstrate the effectiveness of MS in stabilizing pharmaceutical glasses. For example, oxidized porous silicon was used to stabilize indomethacin;^16^ the loaded indomethacin was stable at 40 °C/75% relative humidity (RH) for over six months, whereas pure indomethacin recrystallized after one month. The physical stability of vortioxetine glass loaded in three types of MS, namely MCM, SBA, and MCF, were compared.^17^ The results revealed that the pure drug recrystallized in 1 day under 30 °C/56% RH, whereas the loaded drug exhibited better physical stability for 1 week. Notably, SBA provided the highest stability over three months.

In this study, the effect of MS addition on the physical stability of overloaded celecoxib (CEL) glass is investigated with a focus on the effect of pore size. Although CEL glass has been reported to be stabilized using a diameter of 2.5–3.7 μm and pore size of 23 nm,^18^ the detailed mechanism of the stabilization effect is still unclear. If surface adsorption and confinement effects are the only stabilization mechanisms, then MS cannot stabilize overloaded drugs. However, we demonstrated that the stabilization effect also worked for drug molecules outside of the pores. The stabilization of the overloaded CEL glass is discussed to provide clear guidance for the selection of mesoporous materials as pharmaceutical excipients with an emphasis on the effect of pore size.

## 2 Materials

CEL (Form III) was purchased from the Tokyo Chemical Industry (Tokyo, Japan) and used without further purification. MS (Sylysia350 and Sylysia730) was obtained from Fuji Silysia Chemical (Kasugai, Japan). The physical properties of MS are presented in Table 1.

**Table 1:** Physical Properties of MS

MS	surface Area (m^2^/g)	pore size (nm)	pore volume (mL/g)	particle size (μm)
Sylysia 350	300	21	1.6	4
Sylysia 730	700	2.5	0.44	4

### 2.1 Preparation of CEL/MS Physical Mixtures

CEL/MS binary mixtures were prepared by carefully mixing CEL and MS for 20 min in various ratios using a mortar and pestle for facilitating homogeneous mixing and loading of CEL into pores.^19^ Then, the samples were sieved using a 500-μm screen, melted at 170 °C on a hot plate, and then cooled by allowing them to rest at 25 °C, resulting in the formation of the glass state. The mixing ratios are expressed as the proportion of the MS in this study. For instance, the mixture of CEL and MS with a weight ratio of 25:75 is expressed as a mixture with 75% MS. Sylysia350 and Sylysia730 are abbreviated as SYL350 and SYL730, respectively.

### 2.2 Evaluation of Molecular States Using Differential Scanning Calorimetry (DSC)

The molecular mobility of pure CEL and its mixtures with SYL730 or SYL350 were determined using DSC (Q2000, TA Instruments, New Castle, DE, USA). The instrument was calibrated using indium and sapphire, and dry nitrogen was supplied as an inert gas at a flow rate of 50 mL/min. Approximately 5 mg of each sample was sealed in an aluminum pan and heated to 180 °C at a heating rate of 10 °C/min for melting. To observe the cold crystallization behavior of CEL, the melt was cooled to −50 °C at a rate of 10 °C/min to induce nucleation.^19^ The sample was then heated at a rate of 10 °C/min to observe the glass transition, cold crystallization, and melting behaviors. Three independent samples of each composition were evaluated.

### 2.3 Isothermal Crystallization of CEL Glass on DSC

The influence of MS on the isothermal crystallization of the CEL glass was evaluated at 95 °C. Approximately 5 mg of CEL or its physical mixture with MS was sealed in aluminum pans and heated to 180 °C at a heating rate of 10 °C/min for melting. The samples were then cooled to −20 °C at 10 °C/min, followed by heating to 95 °C at a rate of 10 °C/min, and annealed at that temperature for predetermined periods. The annealing for over 6 h was conducted in a temperature-controlled oven at 95 °C. The samples were then heated from 25 °C at 10 °C/min to observe the glass transition behavior. The remaining glass fraction of the annealed samples was determined by using the heat capacity change at the glass transition temperature (Δ*C*_p_). Three independent samples were analyzed for each annealing period.

### 2.4 Determination of the Size of the Cooperatively Rearranging Region (CRR)

The size of the CRR was determined by DSC in temperature-modulated mode. Approximately 5 mg of CEL and its physical mixture with MS were sealed in Tzero pans and heated to 180 °C at a rate of 10 °C/min for melting, followed by cooling at 10 °C/min to −50 °C. Then, the samples were heated at 2 °C/min under modulation conditions with an amplitude of 0.5 °C and a period of 60 s. Three independent samples were analyzed for each composition. The CRR size (*L*) was determined using the following equation:^20^
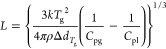
1where ρ and Δ*d*_*T*g_ are the density and half of the glass transition width, respectively; *T*_g_ is the onset glass transition temperature; *k* is Boltzmann’s constant; *C*_pg_ and *C*_pl_ are the specific heat capacities of the glass and supercooled liquid, respectively. The specific heat capacities were determined using a previously established protocol.^21^ Briefly, 10 mg of quenched glass was evaluated using Tzero pans in temperature-modulated mode, and the experiments were repeated 10 times to obtain the mean values. *C*_pg_ and *C*_pl_ were determined to be 1.62 and 2.07 J/(g°C), respectively. The glass transition width was determined from the difference between the onset and end points of *T*_g._

### 2.5 Evaluation of Molecular Mobility on Broadband Dielectric Spectroscopy (BDS)

Broadband dielectric measurements of pure CEL and its mixtures with MS were performed by using a Novo-Control GMBH Alpha dielectric spectrometer (Montabaur, Germany). The sample temperature was controlled by a Quattro temperature controller with temperature stability better than 0.2 °C. The samples were melted on a stainless-steel sample stage using a hot plate heated to 170 °C for 10 min and then quenched under ambient temperature to obtain the glass samples. Thickness of the samples was controlled to 0.1 mm by inserting silica spacer fibers between the stainless-steel plates. The samples with the stage were immediately transferred to the instrument and heated at 190 °C for 1 h to remove residual moisture under a flow of nitrogen gas. The dielectric spectra were acquired in a temperature range from −120 to 140 °C, with an interval of 4 °C (−120 to 0 °C) or 2 °C (0 to 140 °C) in a frequency range from 10^–2^ to 10^7^ Hz. All measurements were repeated twice to confirm reproducibility. The data obtained at each temperature were analyzed by fitting the spectra to the Havriliak–Negami (HN) equation as follows:^18,22,23^
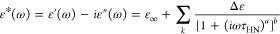
2Here, ε*(ω) is the complex permittivity; ε*’* (ω) and ε*”*(ω) are the real and imaginary parts, respectively, of the complex permittivity; ε_∞_ is the high-frequency limit permittivity; Δε is the relaxation strength; τ_HN_ is the relaxation time; and *a* and *b* are exponents of the relaxation processes. ω is equal to 2π*f*, where *f* denotes frequency. σ_dc_ is the DC-conductivity, and ε_0_ is the permittivity of a vacuum. The α relaxation time τ_*a*_ was determined using the following equation:^18,22,23^
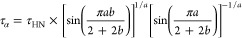
3

### 2.6 Isothermal Crystallization of CEL Glass on BDS

BDS was also used to investigate the isothermal crystallization of the glass samples at 95 °C. The isothermal dielectric response of the CEL glass was measured continuously at 600 s intervals. The frequency range in which the α relaxation peak appeared was investigated. The crystallization process was monitored by the intensity of the real (ε′) part of the complex dielectric permittivity because basically, only the glass phase contributed to the dielectric response.^24^ Therefore, the relative permittivity (ε′_N_) of CEL can be calculated using the normalized change in dielectric dispersion. The equation is as follows:
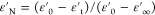
4where ε′_0_ is the initial static dielectric permittivity, ε′_∞_ is the long-time limiting value, and ε′_*t*_ is the value at time *t*. ε′_N_ is almost analogous to crystallinity; however, the calculated values do not have strong physical meaning, as each fraction has different strengths of dipole moment.

### 2.7 X-ray Powder Diffraction (XRPD)

The samples used for BDS and DSC measurements were collected after evaluation and subjected to XRPD analysis. Data were obtained on a Rigaku RINT Ultima X-ray diffraction system (Rigaku Denki, Tokyo, Japan) with Cu Kα radiation. The voltage and current were set to 40 kV and 40 mA, respectively. Data were acquired at a scan rate of 2 °C/min at 0.02° intervals.

### 2.8 Density Measurement

The true density of the CEL glass was measured by using an AccuPyc II gas pycnometer (Micromeritics, Norcross, GA, USA) using helium gas. Approximately 0.5 g of the sample was melted on a hot plate and then cooled under an ambient atmosphere. Subsequently, the obtained glass pellet was ground using a mortar and pestle and dried under a vacuum at 40 °C for 30 min. The powder was confirmed to be in a completely glassy state by XRPD and DSC. The measurements were repeated ten times to obtain the mean value.

## 3 Results

### 3.1 Discrimination of Molecular State of CEL Glass Using DSC

**Figure 1:**
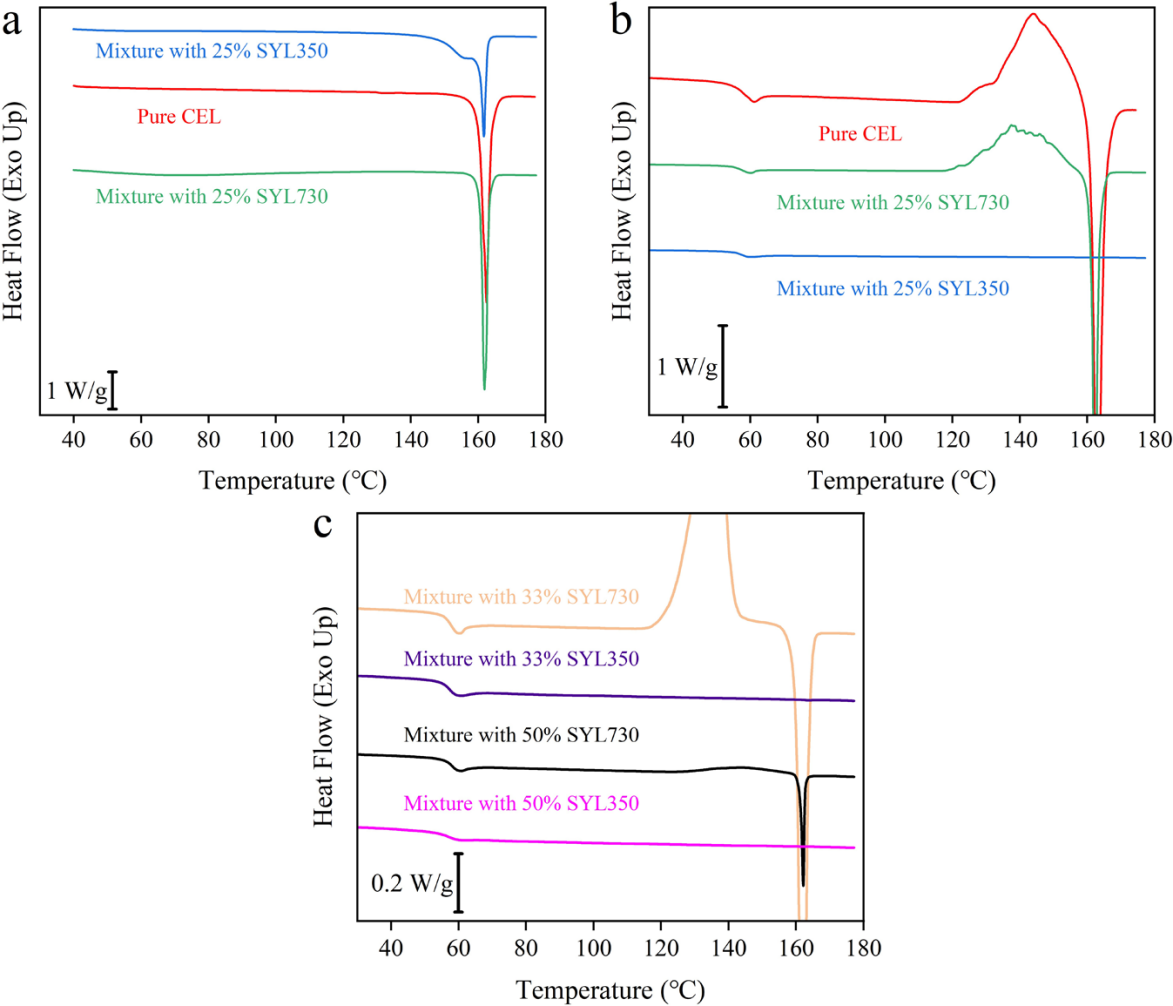
(a) First and (b) second heating DSC curves for pure CEL, its mixture with 25% SYL350, and its mixture with 25% SYL730. After the first heating, the samples were cooled to −50 °C to induce nucleation. (c) Second heating curves for the CEL mixtures with 33 or 50% MS.

Figure 1a,b shows the first and the second DSC heating curves for pure CEL and its physical mixtures with 25% MS, respectively. At this mixing ratio, the amount of CEL exceeded the pore capacity (i.e., overloaded) of both types of MS. The melting behavior of the mixture with SYL730 was similar to that of pure CEL during the first heating; however, the melting peak split into two for the mixture with SYL350. CEL is expected to penetrate the pores during the grinding.^25,26^ The appearance of the fraction that melted at a lower temperature can be explained by the confinement effect of the pores, as described by the Gibbs–Thomson equation.^27^ Presumably, the pore size of SYL730 was too small to allow the crystalline CEL to penetrate during the grinding.

The melted samples were cooled to −50 °C and then heated again to acquire the second heating curves (Figure 1b), where the onset *Τ*_g_ values were ca. 58 °C for all samples. A cold crystallization peak appeared for pure CEL and the mixture with 25% SYL730, whereas it was not observed for the mixture with 25% SYL350. This observation indicates that SYL350 has a stronger stabilization effect on CEL, despite its smaller surface area compared to that of SYL730. The proportion of MS was increased to determine the effect of the mixing ratio on the stabilization of the CEL glass (Figure 1c). The thermal behaviors for the mixture with SYL350 remained almost the same, except for Δ*C*_p_, which decreased with an increasing MS amount. For the mixture with SYL730, in addition to the decrease in Δ*C*_p_, the cold crystallization peak became smaller with increasing MS amount. However, small crystallization and following melting peaks were observed even for the mixture containing 50% SYL730.

**Figure 2:**
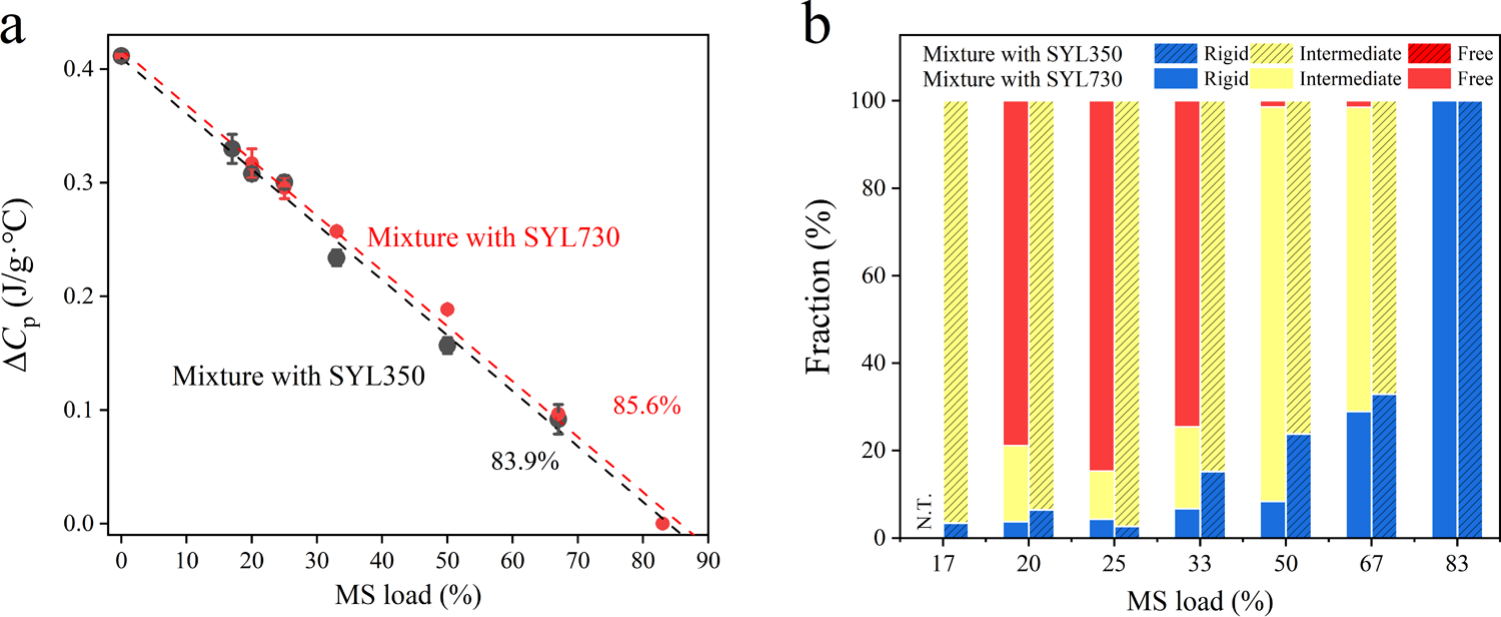
(a) Changes in Δ*C*_p_ (expressed as per gram of the mixture) as a function of the loading proportion of MS. (b) Rigid, intermediate, and free fractions of CEL molecules in mixtures with SYL350 or SYL730. NT: not tested.

Δ*C*_p_ is assumed to be proportional to the mobile glass fraction of CEL. The dependence of Δ*C*_p_ of each mixture on the proportion of MS is shown in Figure 2a. Linear extrapolation of each data set provided the required amount of MS to erase the glass transition of CEL as 83.9 and 85.6% for SYL350 and SYL730, respectively. The disappearance of the glass transition behavior can be explained by the strong interaction of CEL molecules with the MS surface. In the field of polymer chemistry, the amorphous part where the glass transition cannot be observed is called the rigid amorphous.^28^ Thus, the invisible CEL glass in this study is hereafter referred to as the rigid fraction. The rigid fraction may be expected to be proportional to the surface area of the MS, which includes both surfaces inside and outside pores. However, it was slightly larger for SYL350 than for SYL730, indicating that the pores were not filled completely, at least for SYL730.

Although the amount of the rigid fraction was not significantly different for both MS, their stabilization effects were completely different, as shown in Figure 1b,c, suggesting the important roles of the mobile fraction on the physical stability of CEL glass. For example, in the presence of 33% MS, the amount of rigid fraction was 15.2 and 6.7% for SYL350 and SYL730, respectively. Most of the CEL glass remained mobile in the presence of both MS. However, cold crystallization was not observed in the mixture with SYL350 during DSC heating, whereas most of the mobile CEL crystallized in the mixture with SYL730. The fraction that exhibited cold crystallization during DSC heating was assumed to have a molecular mobility similar to that of pure CEL and is hereafter termed the free fraction. The remaining fraction, which exhibits glass transition behavior but is unable to crystallize during heating, is called the intermediate fraction. The free fraction was calculated from the cold crystallization enthalpy. Figure 2b and Table 2 show the classification of each fraction for various mixing ratios of MS. In the mixture with SYL350, most CEL molecules, except for the rigid fraction, existed as the intermediate fraction, regardless of the mixing ratios. In contrast, most CEL molecules behaved as free fractions in the mixture with SYL730.

**Table 2:** Calculated Rigid and Free Fractions of CEL Glass in the Mixture with SYL350 or SYL730

	mixture with SYL350		mixture with SYL730	
MS load (%)	rigid (%)	free (%)	rigid (%)	free (%)
17	3.42 ± 3.77	0.00	N.T.	N.T.
20	6.47 ± 1.75	0.00	3.73 ± 3.93	78.9 ± 14.2
25	2.67 ± 1.90	0.00	4.28 ± 3.07	84.7 ± 6.0
33	15.2 ± 2.5	0.00	6.69 ± 0.78	74.6 ± 9.7
50	23.8 ± 3.4	0.00	8.36 ± 1.13	1.42 ± 2.47
67	32.4 ± 9.6	0.00	28.9 ± 0.6	1.49 ± 1.29
83	100	0	100	0

The absence of proportionality of the rigid fraction to the surface area of the MS indicated imperfect penetration of the CEL molecules into the small pores.^29^ Using the density of the CEL glass (1.41 g/cm^3^), the required amounts of SYL350 and SYL730 to completely capture the CEL molecules into the pores were calculated to be 31 and 61%, respectively, based on the pore volume of MS. The strong stabilization effect is expected on molecules adsorbed as a monolayer.^30^ The adsorbed area per CEL molecule was calculated as ca. 0.59 nm^2^ using the bulk density value under a simple assumption of the cubic shape of CEL molecules. Given that the surface of MS is densely packed with monolayered CEL, SYL350, and SYL730 theoretically can load 5.1 × 10^20^ and 1.2 × 10^21^ CEL molecules per gram, respectively; thus, the required amounts of MS to offer monolayer adsorption sites for all CEL molecules are 76 and 57%, respectively. In the experiment, the required amounts of MS for changing all CEL molecules into the rigid fraction were 83.9 and 85.6%, respectively. Based on this calculation, approximately 9% and 33% of the pores in SYL350 and SYL730, respectively, were unfilled. Because this calculation includes some assumptions, such as the use of the bulk density value to estimate the adsorption area per CEL molecule, SYL350 may be almost filled, whereas this is not true for SYL730. Approximately one-third of the pores of SYL730 were likely to be unfilled.

### 3.2 Investigation of Molecular Mobility Using BDS

**Figure 3:**
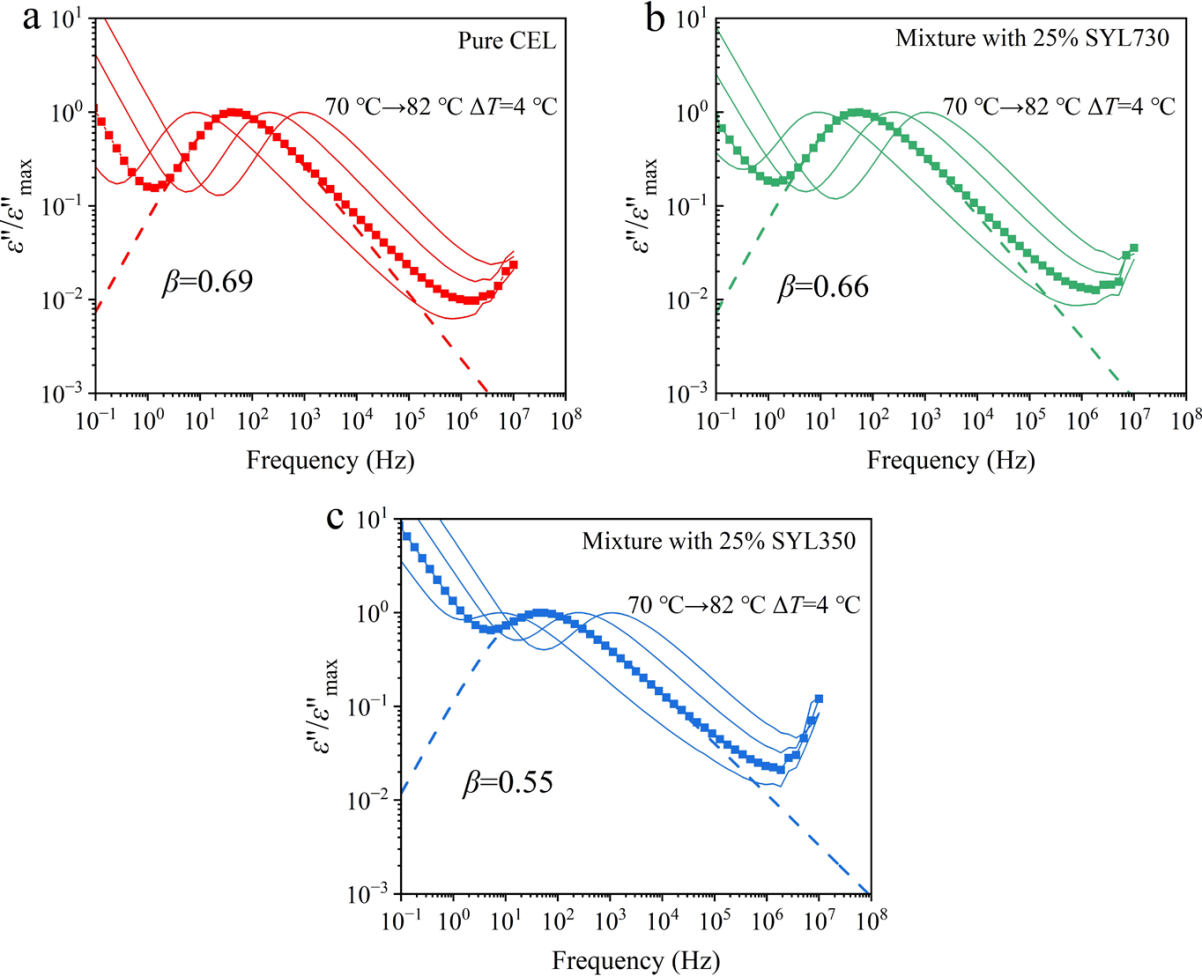
Normalized dielectric spectra of (a) pure CEL, (b) its mixture with 25% SYL730, and (c) its mixture with 25% SYL350, respectively. The KWW fitting at 74 °C is represented by break lines. ε″_max_ is the maximum value of the α relaxation peak.

**Figure 4:**
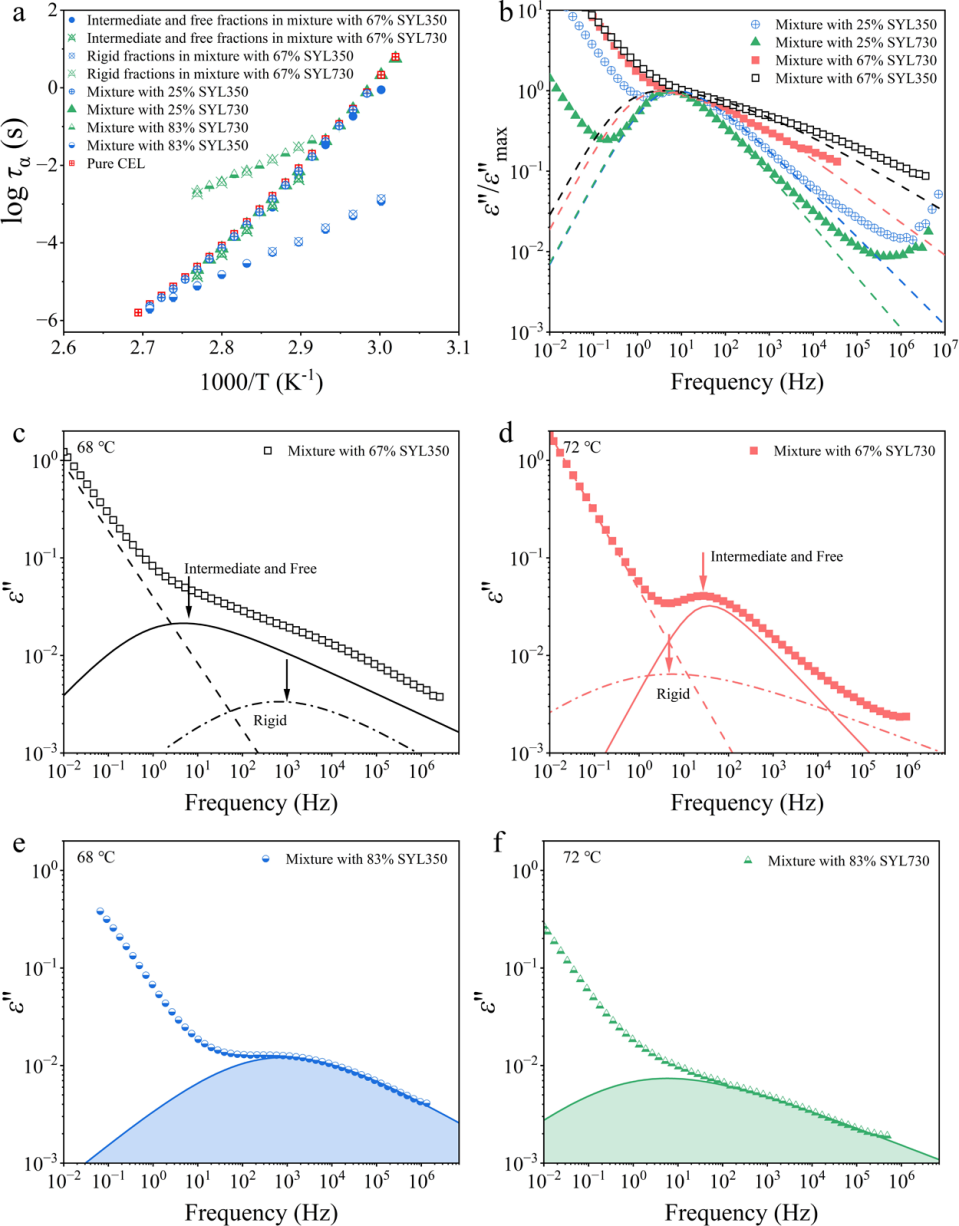
(a) Relaxation time (τ_α_) of CEL glass and its mixtures with various amounts of SYL350 or SYL730; all fractions exist for the mixtures with 25% MS but the amount of rigid fraction is negligible; intermediate and rigid fractions exist for 67% MS, and only rigid fraction is available for 83% MS. (b) Normalized dielectric spectra and KWW fitting (as presented by break lines) of the mixtures with 25 or 67% of MS at 70 °C. (c) An example of deconvolution of dielectric loss spectra of the mixture with 67% SYL350 at 68 °C into rigid and Intermediate + free fractions. (d) An example of deconvolution of dielectric loss spectra of the mixture with 67% SYL730 at 72 °C. (e) Dielectric loss spectra of a mixture with 83% SYL350 at 68 °C (1000/*T* = 2.93). (f) A dielectric loss spectra of a mixture with 83% SYL730 at 72 °C (1000/*T* = 2.90).

Figure 3 shows the normalized dielectric spectra obtained by BDS measurements of the CEL glass and its mixture with 25% MS, where all fractions are expected to coexist. The intermediate fraction is dominant in the mixture with SYL350, whereas the free fraction dominates, but the intermediate fraction should also have a contribution in the mixture with SYL730. All spectra fit well with the superposition of the HN function (Figures 3a–c). Figure 4a shows the τ_α_ value as a function of temperature, where the Vogel–Fulcher–Tammann (VFT) equation was used to fit the data.^31^
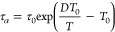
5where τ_0_ is the time scale of the vibrational motion, *T* is the temperature, *T*_0_ is the temperature analogous to the Kauzmann temperature, and *D* is a strength parameter. The data for pure CEL and its mixtures with 25% MS could be fitted by similar VFT functions, and the fitting parameters are reported in the Supporting Information. The shape of the α relaxation peak provides information on the distribution of τ_α_, which can be described by the β parameter in the Kohlrausch–Williams–Watts (KWW) function.^31^
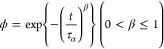
6where *Φ* and *t* are the remaining fractions of the excess enthalpy with reference to the extrapolation of the supercooled liquid to a lower temperature, and time, respectively. A large β value indicates homogeneity of the relaxation time, which makes the dielectric spectral peaks symmetric. The fitting of the BDS data revealed that pure CEL and the mixture with 25% SYL730 shared similar β values of 0.69 and 0.66, respectively. However, the β for the mixture with 25% SYL350 was 0.55, suggesting that the addition of SYL350 made the molecular mobility of CEL heterogeneous, most likely due to a large proportion of the intermediate fraction.

Figure 4b shows the normalized dielectric spectra of the mixtures with 67% MS, where those for 25% mixtures are retained as a comparison. For the mixtures with 67% MS, the presence of two components must be assumed for successful fitting. Thus, τ_a_ values were obtained by applying deconvolution to the spectra (Figure 4c,d). As almost no free fraction exists for both (Figure 2b), the obtained two τ_a_ values are likely to be assigned to those of intermediate and rigid fractions. As presented in Figure 4a, one of the mean τ_α_ of CEL with SYL350 and SYL730 was both comparable to that of pure CEL. This observation aligns with previously reported results for the CEL mixtures with SYL244FP, which has a pore size of 23 nm, ranging from 9 to 45%.^18^ Furthermore, their results reported that the β values of the mixtures decreased from 0.65 to 0.45 with increasing amounts of MS. Our observations also revealed a widening of the dielectric spectra, which suggested a decrease in β values. The β values of the intermediate fraction in the mixture with 67% SYL350 and SYL730 were determined to be 0.31 and 0.57, respectively.

The mobility of the rigid fraction was investigated using mixtures with 83% MS (Figure 4e,f). The β values for the rigid fraction in the mixture with 67% SYL350 and 67% SYL730 were 0.30 and 0.25, respectively. The mean τ_α_ values (Figure 4a) were influenced differently by the addition of MS. The rigid fraction in SYL350 had higher molecular mobility relative to free and intermediate fractions, whereas the opposite trend was observed in the presence of SYL730. Thus, molecular mobility of the rigid fraction was likely to be influenced by pore size. Although a decrease in molecular mobility is easy to imagine considering its interaction with the MS wall, an increase in mobility appears to be unnatural. However, both effects have been observed in previous studies. The mobility of naproxen glass encapsulated in MS with a pore size of 5.9 nm exhibited two α relaxation processes: a slower relaxation belongs to molecules interacting with the pore surface and a faster relaxation that happens in the center, whereas such multimodal processes were not the observed for the naproxen glasses in 2.4 or 3.2 nm-pore.^32^ On the other hand, ibuprofen glass showed faster mobility inside MCM with pore sizes of 3.5 and 11.6 nm,^33^ which may be explained by a decrease in the dimension of molecular motion.^34−36^ As for clotrimazole glass, density functional theory simulation proved that the high flexibility of the silica surface silanols made the glass molecules highly mobile in the MSU-H having 8.5 nm pores.^37^ Thus, an increase in molecular mobility in nanopores has also been a general observation.

### 3.3 Size of CRR of CEL Glass

**Figure 5:**
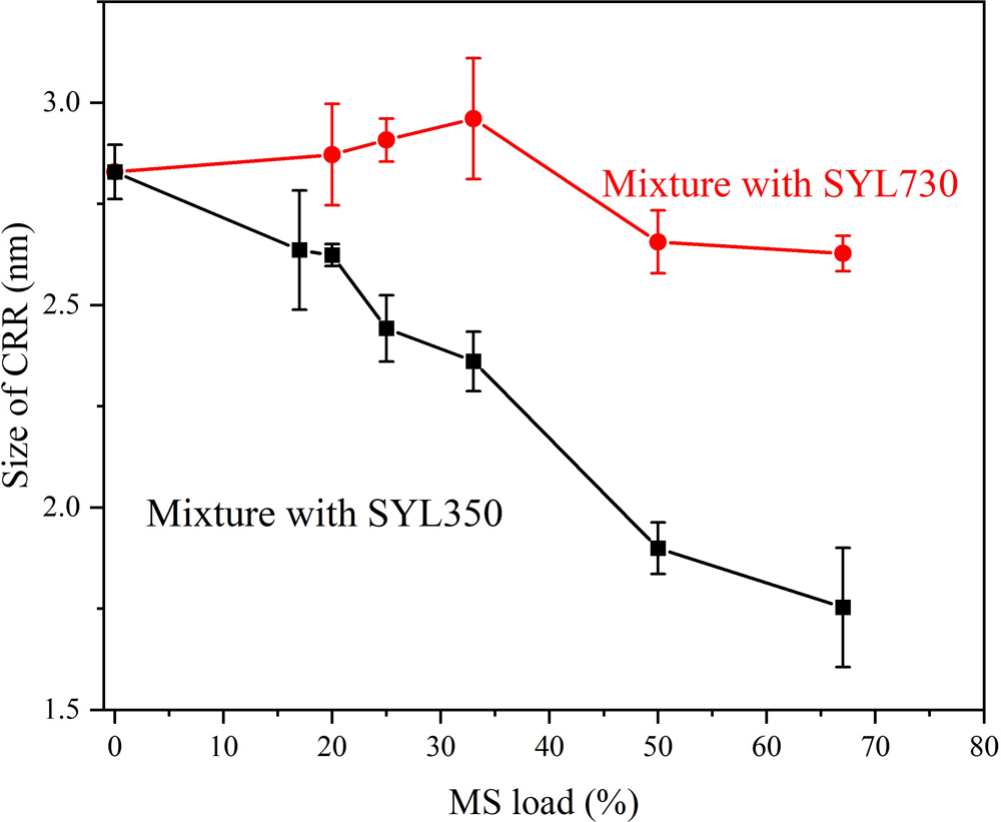
Sizes of the CRR of CEL glass in its mixture with SYL350 or SYL730.

Figure 5 shows the effect of MS addition on the CRR size of the CEL glass. Because *Τ*_g_ was the same for the intermediate and free fractions, this estimation was the average for the two fractions. Upon mixing with SYL730, it remained almost constant up to 33% MS, and decreased beyond this ratio. This observation corresponded to a change in the intermediate/free fraction ratio (Figure 2b). Mixing with SYL350 significantly reduced the size of the CRR, even in small amounts. This analysis suggests that the intermediate fraction has a CRR smaller than that of the bulk molecules.

### 3.4 Effect of MS on the Physical Stability of CEL Glass

**Figure 6:**
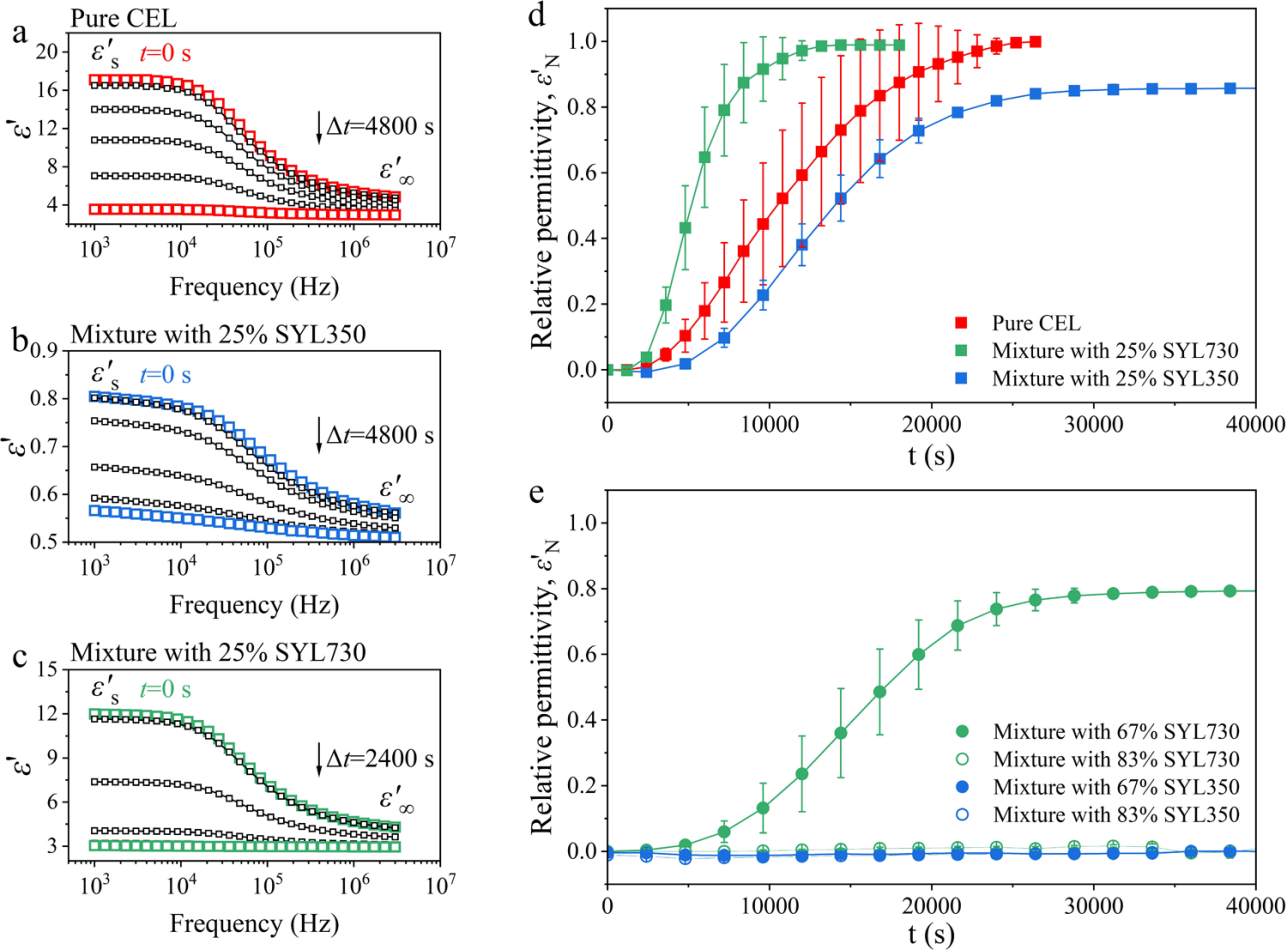
Decrease of ε*′*_s_ during isothermal annealing of the glass samples at 95 °C: (a) pure CEL, (b) mixture with 25% SYL350, and (c) mixture with 25% SYL730. (d) and (e) ε′_N_ of mixture with different amounts of MS determined by eq 4, as a function of time.

The effect of MS addition on the physical stability of the CEL glass at 95 °C was investigated using BDS and DSC. Representative BDS spectra of the three samples are presented in Figure 6a–c. Unlike in DSC, a rigid fraction was detectable in the BDS study. However, the weak intensity of static permittivity (ε′_s_) in the mixture with SYL350 suggested a reduction in the dipole moment of the intermediate and rigid fractions. During annealing, the CEL glass in all samples showed recrystallization, as reflected by the decreasing ε′_s_. However, for the mixtures with MS, the decrease of ε′_s_ halted before reaching 100% crystallinity. The evolution curves of ε*′*_N_ for pure CEL and its mixtures with MS are shown in Figure 6d. The ε′_N_ values of mixtures with 25% SYL350 and SYL730 stopped growing at approximately 86 and 99%, respectively. As the rigid fraction is not expected to crystallize, the ε′_N_ does not reach zero. However, the remaining is larger than the proportion of the rigid fraction for the mixture with SYL350, suggesting that a part of the intermediate fraction was not likely to be crystallized. The time to reach 50% ε′_N_ (*t*_1/2_) of the mixture with 25% SYL350 was the longest at 14400 s, followed by pure CEL (10800 s) and the mixture with 25% SYL730 (5400 s). Thus, the addition of SYL350 retarded the crystallization of CEL glass, whereas the addition of SYL730 accelerated it when a free fraction exists. The physical stability of CEL was enhanced by increasing the amount of MS. The crystallization of CEL was slower than that of pure CEL when it was mixed with 67% SYL730, and the crystallization was not observed with 67% SYL350 (Figure 6e). When 83% MS was added, crystallization never proceeded for both types of MSs.

**Figure 7:**
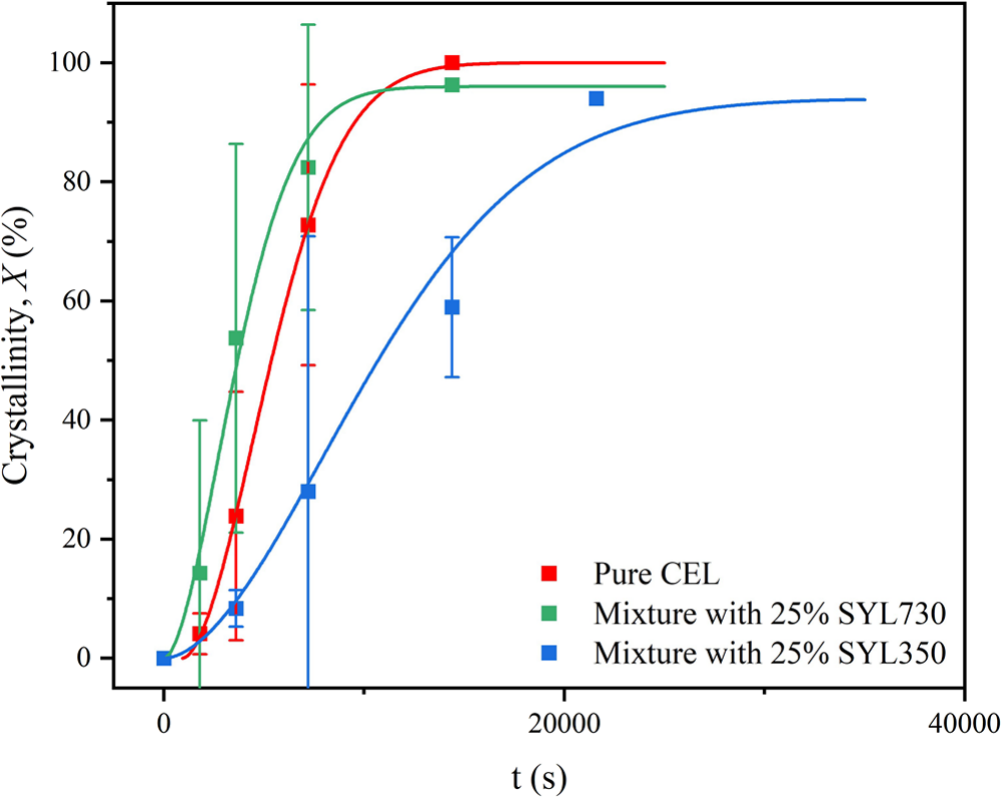
Evolution of crystallinity of pure CEL, mixture with 25% SYL350, and mixture with 25% SYL730 at 95 °C, as determined by DSC.

DSC was also used for investigating the isothermal crystallization of the mixtures with 25% MS at 95 °C. The crystallization curves are shown in Figure 7. The crystallinity (*X*) of CEL was calculated by using Δ*C*_p_ under the assumption that it is proportional to the amount of the remaining amorphous phase. The data were fitted using the Avrami–Erofeev equation:
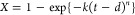
7where *k* and *d* are the crystallization rate constant and induction time, respectively. *n* is the Avrami exponent, which reflects the dimensions of the crystal growth and nucleation mechanisms.

The crystallization curves were very similar to those obtained from the BDS measurements (Figure 6d); that is, the addition of SYL350 and SYL730 retarded and accelerated crystallization, respectively, and a part of the amorphous phase remained uncrystallized. The uncrystallized fraction as revealed by DSC was 6 and 4%, respectively, in the presence of SYL350 and SYL730.

The kinetic parameters obtained are given in Table 3. The uncrystallized fraction was ignored in the fitting procedure of the Avrami equation. The Avrami exponents obtained were approximately 2 for both pure CEL and its mixture with MS, suggesting that the presence of MS did not influence the crystallization mechanism. This may be due to the fiber-like growth properties of the CEL crystals^38^ that can fit the pore structure. The results show that *k* was smaller and larger in the presence of SYL350 and SYL730, respectively, than in the presence of pure CEL. As shown next, a small amount of metastable form III was included after crystallization with SYL350. Thus, the slow crystallization may partially be responsible for the appearance of a different crystal form. The kinetic parameters obtained could be understood as those for the stable form I. As the crystallization of CEL glass frequently proceeds into mixtures of form I and III,^22^ these forms are likely to grow independently, that is, no interference is anticipated during the crystallization. Moreover, both stable and metastable forms (I and III) of pure CEL have almost the same growth rate at 95 °C.^38^ Thus, the presence of a small amount of form III does not have an impact on the kinetic analysis.

**Table 3:** Kinetic Parameters for the Isothermal Crystallization of Pure CEL Glass and the Mixture with 25% SYL350 or SYL730 from DSC

sample	*k* (s^–1^)	*d* (s)	*n*
CEL	1.59 × 10**^–7^**	907	1.8
mixture with 25% SYL350	4.64 × 10**^–8^**	0	1.8
mixture with 25% SYL730	4.06 × 10**^–7^**	0	1.8

### 3.5 Physical Characterization of Recrystallized CEL

**Figure 8:**
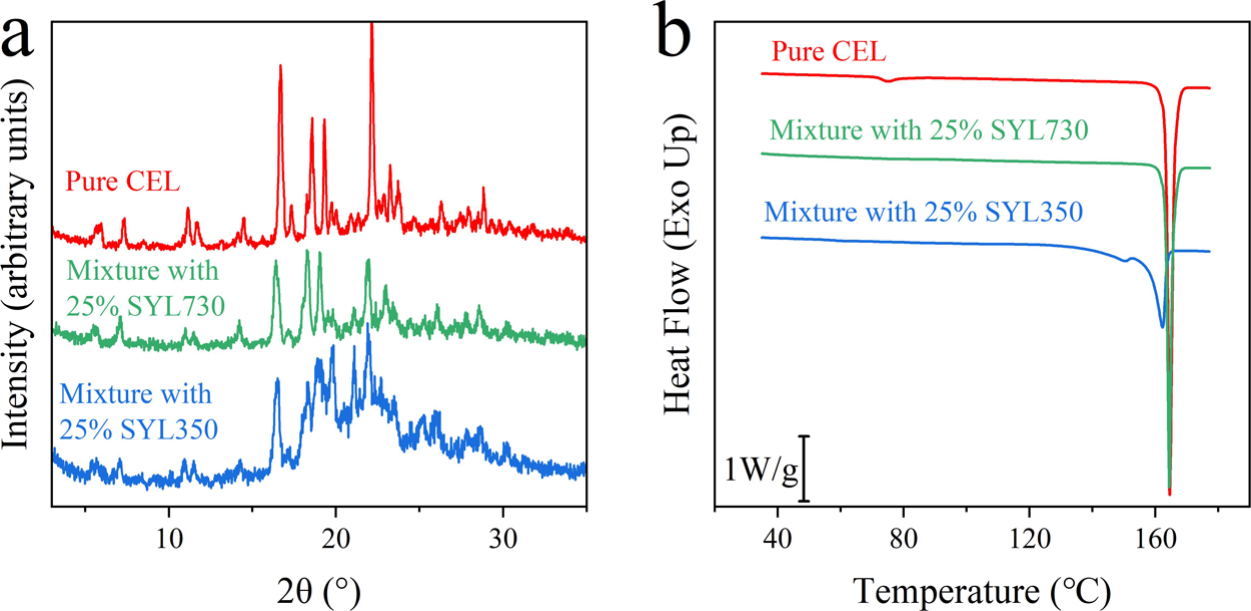
(a) XRPD patterns for pure CEL and its mixture with 25% MS after the isothermal crystallization study at 95 °C by using BDS. (b) DSC curves for pure CEL and its mixture with MS after 14400 s (4 h) of annealing at 95 °C.

The recrystallized samples at 95 °C in the BDS study were subjected to XRPD analysis (Figure 8a). CEL can exist in five crystal forms (Form I, II, III, IV, and V),^22,39,40^ where the most stable form at 95 °C is the Form I. The XRPD pattern of pure CEL was characteristic of Form I and represented by peaks at 5.5°, 5.7°, 7.2°, and 16.6°. These peaks were observed in the XRPD patterns of the mixtures recrystallized in the presence of SYL730 or SYL350. In addition, the characteristic peak of Form III (19.2°) was observed for the mixture with SYL350.

Figure 8b shows the DSC curves of the recrystallized CEL in the presence of MS. The melting peak of Form I (ca. 164 °C) was observed for pure CEL and its mixture with 25% SYL730. Two endothermic peaks were observed at ca. 151 and 162 °C for the mixture with 25% SYL350. These peaks can be attributed to Forms III and I, respectively, under the influence of pores, as observed for the physical mixture (Figure 1). A small endothermic peak with an enthalpy value of about 2.6 J/g was observed for pure CEL at about 75 °C in a reproducible manner. Because a small endothermic peak indicates an enantiotropic polymorphic transformation,^41^ traces of other crystal forms might exist in the recrystallized sample.

## 4 Discussion

### 4.1 Molecular Mobility of CEL Molecules as Indicated by BDS measurements

In this study, CEL glass was divided into three types based on its thermal behavior during DSC heating. A comparison with the BDS results provided further insights. The mobility of free and intermediate fractions was similar in the BDS measurements, as proven by a similar mean τ_α_ (Figure 4a). The rigid fractions are likely to behave in opposite ways depending on the pore size. When the pore size is comparable to or smaller than CRR, the mobility of the rigid fraction seemed to be suppressed. However, the mobility appeared to be enhanced in larger pores, presumably because of the easiness of exchange of molecules inside and outside pores. In literature, water molecules inside the graphene slab, of which the pore size was 3.1 nm, were reported to exhibit fast diffusion.^42^ The motion parallel to the pore wall was assumed to be faster than that perpendicular to the surface, and the diffusion mostly occurs in a parallel direction to the wall in a confined space.^43^ However, the diffusion was slower in the pore of 0.7 nm, most likely because of the suppression of translational motion.^43^ According to a dielectric relaxation spectroscopy study of ibuprofen glass in MCM-41, whose diameter is 3.6 nm, higher molecular mobility was likely to be retained near and below the *T*_g_ compared to that of bulk ibuprofen,^44^ which was also verified by magic-angle spinning and pulsed-field gradient NMR techniques.^45^ In a study of flufenamic acid glass, the presence of a liquid-like layer that has high molecular mobility on the nanopore surface was proposed based on investigation using ^19^F NMR spectroscopy.^14^

Presumably, the high stability of the rigid fraction is not related to molecular mobility but to geometrical restriction in the pores. In the isothermal crystallization study by BDS (Figure 6), the ε′_N_ of the mixtures with 25% SLY350 and SYL730 reached 86 and 99%, respectively, whereas their rigid fractions were only 2.7 and 4.3%, respectively, in the DSC measurements. The higher final ε′_N_ of the mixture with SYL730, compared to that expected based on the amount of the rigid fraction, may be understood as within experimental error; however, as the disagreement for the mixture with SYL350 was quite large, an additional explanation is required for the apparent low ε′_N_. One possible explanation is the contribution of the crystallized part to the dielectric spectra. The decrease in dielectric loss was caused by a reduction in active dipoles after crystallization.^46^ However, the crystal microstructure can significantly influence the dielectric loss.^47^ The remaining dielectric loss of the mixture with 25% SYL350 could have originated from the orientation of the associated dipole located in defects, such as dislocations and vacancies, which exist in the form of loose crystallization in larger pores.^48^ In fact, the final crystallinity in the DSC measurement reached 94% (Figure 7).

The crystallization of CEL with 67% SYL350 was completely inhibited, whereas it was significantly retarded with 67% SYL730. Almost no free fraction was expected to exist at this mixing ratio for both MS. The ε′_N_ reached 80% in the presence of 67% SYL730, which is smaller than the amounts of intermediate and free fraction (66%). This discrepancy may be explained by the weak dipole moment of the rigid fraction.

### 4.2 Influence of Pore Size of MS on the Stabilization of CEL Glass

The physical stability of the CEL glass showed opposing trends, depending on the pore size in the presence of 25% MS. The crystallization of CEL glass with 25% SYL730 was faster than that of pure CEL, whereas 25% SYL350 delayed the crystallization. Although SYL730 had a larger surface area than SYL350, its smaller pores did not allow easy penetration of CEL molecules, resulting in a similar amount of rigid fraction in both MS. Considering the size of the CEL molecules, the number of molecules per cross-sectional area of the pores is estimated to be ca. 750 and 11 for SYL350 and SYL730, respectively. The most striking difference in the mobility of the overloaded CEL molecules was the presence of a large intermediate fraction in the mixtures with SYL350. It is difficult to explain the delayed crystallization of CEL in the presence of SYL350 solely based on molecular mobility, as the mobility of free and intermediate fractions was similar (Figure 4a). Thus, the steric hindrance of the nanopores for crystal growth should also be responsible for stabilization, although a large fraction of CEL molecules existed outside the pore. Accelerated crystallization in the presence of SYL730 was an interesting observation. In the literature, both physical stabilization and destabilization of pharmaceutical glasses have been reported for MS, suggesting a complicated influence on the stability. The stabilization mechanism includes the interaction of the drug with the MS surface and the confinement effect of the pores. In addition, MS seems to have a destabilizing effect, which may be explained by its templating property. Notably, the same MS can either stabilize or destabilize the system depending on the mixing ratio, as observed for aripiprazole glass.^49^

**Figure 9:**
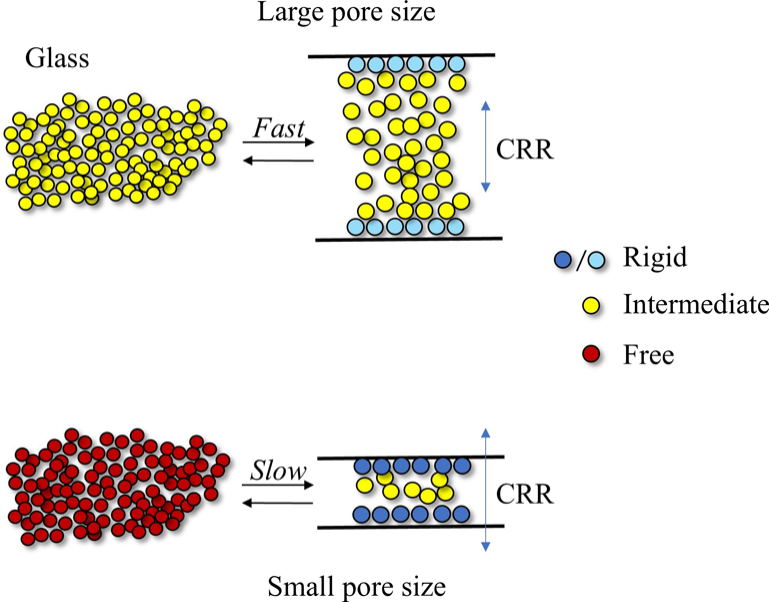
Schematic presentation of the influence of pore sizes on the dynamics of glass molecules. Rigid, intermediate, and free fractions are presented by blue/cyan, yellow, and red, respectively. The rigid fraction exists as monolayers on the wall. It has a higher mobility in SYL350 but a lower mobility in SYL730. The intermediate fraction retains bulk-like mobility and is exchangeable inside and outside pores. The stabilization effect based on the confinement effect is effective for the outside molecules as well as for the inside molecules, as the molecules are easily exchangeable inside/outside pores. When the exchange is slow, no stabilization effect is exerted on the free fraction outside the pores, as observed for SYL730.

The size of the CRR of the CEL glass was smaller than the pore size of SYL350 but comparable with that of SYL730 (Figure 5). Therefore, the size of the CRR may explain the difficulty in the exchange of inside and outside molecules for SYL730. The three fractions of CEL glass and their possible exchange behaviors inside and outside the pores are presented in Figure 9. The CEL molecules near the surface form a rigid fraction. When the pore size is sufficiently larger than the size of the CRR, the molecules inside and outside the pores appear to be easily exchanged. Crystallization was delayed in the presence of SYL350, mainly because of geometrical restrictions, as only molecules outside the pores were allowed to grow into large crystals. The molecules in the pores must “wait″ until they are released outside the pores, which causes a delay in crystal growth. Moreover, nuclei formed outside the pore may diffuse into the pores to inhibit their growth into large crystals. When the pore size is comparable to or smaller than that of the CRR, as in the case of SYL730, the exchange of molecules inside and outside the pores is expected to be extremely slow. No stabilization effects were expected based on the geometrical restriction for the molecules outside the pores.

Previously, the difference in the particle size of MS was found to influence the stabilization effect; simvastatin glass was physically stabilized by mixing it with MS of small particle size.^12^ The MS with a large particle size did not offer a stabilization effect despite having the same pore size of 23 nm, which can be explained in a similar manner. If the particle size is small, then the exchange of molecules is easy because of the large surface area of the particles. If the particle size is too large, then only entrapped molecules near the surface are involved in the stabilization effect. In summary, exchangeable molecules, which can be quantified as intermediate fractions, are likely to play an important role in the physical stabilization of overloaded glass. A sufficiently large pore size and small particle size are required to exert the stabilization effect.

From a practical point of view, the perfect filling of small pores is anticipated to be difficult by any means. In fact, our model calculation revealed that one-third of the pores of SYL730 was assumed to be unfilled even in the mixture with a sufficiently large amount of CEL. If guest molecules penetrate from both sides of a connected pore, the pressure inside the pore increases, which should inhibit further penetration of the guest molecule. If the loading of drug molecules is done under high-temperature conditions, a decrease in temperature should allow additional loading of the guest molecules due to a decrease in pressure in the pores. However, even with this scenario, perfect loading cannot happen as the pore environment is not a vacuum. This is also a reason for the poor stabilization effect of MS with too small pores.

### 4.3 Influence of the Pore Size of MS on the Crystal Form of CEL Glass

Form III is the most stable crystal form for CEL at room temperature, whereas Form I is the most stable at temperatures higher than about 60 °C.^22^ In this study, only Form I appeared in the mixture with 25% SYL730 at 95 °C. In contrast, Form III was likely to be formed in a mixture containing 25% SYL350. Similar observations, where nanoconfinement influenced the form of the recrystallized drug, have been frequently reported. In the case of the crystallization of phenyl salicylate, an unstable (monoclinic) form was preferred in the presence of anodic alumina oxide membranes with 150 nm pores, whereas a stable (orthorhombic) crystal was found in that with 100 nm pores.^50^ When bulk probucol crystallizes into Form I, unstable Form II is found in nanochannels with a 40–120 nm pore size.^51^ This observation revealed the usefulness of mesoporous materials for control of the crystal forms. In addition, our study indicated that a larger pore size is required relative to that of the CRR for the controlled crystallization as well as for the stabilization of the amorphous state. The slow crystallization in the presence of SYL350 may be related to the occurrence of the metastable form, although its amount was small.

## 5 Conclusions

In this study, the stabilization effects of two MS with different pore sizes on the overloaded CEL glass were compared. CEL glass in the presence of MS was classified using DSC into free, intermediate, and rigid fractions based on its molecular mobility. The intermediate fraction appears to play an influential role in the stabilization effect. The MS with a large pore size (SYL350) significantly retarded the crystallization of the CEL glass, most likely because most of the overloaded CEL molecules could exist as an intermediate fraction. In contrast, the crystallization of CEL glass was accelerated in the mixture with SYL730, when the amount of MS was not sufficient, where most of the overloaded CEL glass remained as the free fraction. This difference likely originated from the difference in the ease of exchange of CEL molecules inside and outside of the pores. The pore size of SYL350 was sufficiently larger than the size of CRR of the CEL glass, but it is not true for SYL730, which might affect the exchange dynamics of the CEL molecules. These findings offer valuable insights into the effect of pore size on the stabilization effect of overloaded pharmaceutical glass.

## Supporting Information Available

Comparison of *Τ*_g_, fragility, VFT parameters of pure CEL, and mixture with MS of different concentrations (PDF)

## References

[ref1] Brouwers J.; Brewster M. E.; Augustijns P. Supersaturating Drug Delivery Systems: The Answer to Solubility-Limited Oral Bioavailability?. J. Pharm. Sci. 2009, 98, 2549–2572. 10.1002/jps.21650. 19373886

[ref2] Kawakami K. Modification of Physicochemical Characteristics of Active Pharmaceutical Ingredients and Application of Supersaturatable Dosage Forms for Improving Bioavailability of Poorly Absorbed Drugs. Adv. Drug Delivery Rev. 2012, 64, 480–495. 10.1016/j.addr.2011.10.009. 22265844

[ref3] Newman A.; Knipp G.; Zografi G. Assessing the Performance of Amorphous Solid Dispersions. J. Pharm. Sci. 2012, 101, 1355–1377. 10.1002/jps.23031. 22213468

[ref4] Kawakami K. Theory and Practice of Supersaturatable Formulations for Poorly Soluble Drugs. Ther. Delivery 2015, 6, 339–352. 10.4155/tde.14.116. 25853309

[ref5] Kawakami K. Supersaturation and Crystallization: Non-Equilibrium Dynamics of Amorphous Solid Dispersions for Oral Drug Delivery. Expert Opin. Drug Delivery 2017, 14, 735–743. 10.1080/17425247.2017.1230099. 27598556

[ref6] Qian K. K.; Bogner R. H. Application of Mesoporous Silicon Dioxide and Silicate in Oral Amorphous Drug Delivery Systems. J. Pharm. Sci. 2012, 101, 444–463. 10.1002/jps.22779. 21976048

[ref7] Xu W.; Riikonen J.; Lehto V. P. Mesoporous Systems for Poorly Soluble Drugs. Int. J. Pharm. 2013, 453, 181–197. 10.1016/j.ijpharm.2012.09.008. 22990124

[ref8] Maleki A.; Kettiger H.; Schoubben A.; Rosenholm J. M.; Ambrogi V.; Hamidi M. Mesoporous Silica Materials: From Physico-Chemical Properties to Enhanced Dissolution of Poorly Water-Soluble Drugs. J. Controlled Release 2017, 262, 329–347. 10.1016/j.jconrel.2017.07.047. 28778479

[ref9] Rengarajan G. T.; Enke D.; Steinhart M.; Beiner M. Stabilization of the Amorphous State of Pharmaceuticals in Nanopores. J. Mater. Chem. 2008, 18, 2537–2539. 10.1039/b804266g.

[ref10] Kawakami K. Crystallization Tendency of Pharmaceutical Glasses: Relevance to Compound Properties, Impact of Formulation Process, and Implications for Design of Amorphous Solid Dispersions. Pharmaceutics 2019, 11, 202 10.3390/pharmaceutics11050202. 31052392 PMC6572324

[ref11] Kawakami K.; Harada T.; Miura K.; Yoshihashi Y.; Yonemochi E.; Terada K.; Moriyama H. Relationship between Crystallization Tendencies during Cooling from Melt and Isothermal Storage: Toward a General Understanding of Physical Stability of Pharmaceutical Glasses. Mol. Pharmaceutics 2014, 11, 1835–1843. 10.1021/mp400679m. 24731254

[ref12] Knapik-Kowalczuk J.; Kramarczyk D.; Chmiel K.; Romanova J.; Kawakami K.; Paluch M. Importance of Mesoporous Silica Particle Size in the Stabilization of Amorphous Pharmaceuticals—the Case of Simvastatin. Pharmaceutics 2020, 12, 21–25. 10.3390/pharmaceutics12040384. PMC7238159 32331310

[ref13] Nishiwaki A.; Watanabe A.; Higashi K.; Tozuka Y.; Moribe K.; Yamamoto K. Molecular States of Prednisolone Dispersed in Folded Sheet Mesoporous Silica (FSM-16). Int. J. Pharm. 2009, 378, 17–22. 10.1016/j.ijpharm.2009.05.023. 19465097

[ref14] Nartowski K. P.; Malhotra D.; Hawarden L. E.; Sibik J.; Iuga D.; Zeitler J. A.; Fábián L.; Khimyak Y. Z. 19 F NMR Spectroscopy as a Highly Sensitive Method for the Direct Monitoring of Confined Crystallization within Nanoporous Materials. Angew. Chem., Int. Ed. 2016, 55, 8904–8908. 10.1002/anie.201602936. 27272008

[ref15] Cheng S.; McKenna G. B. Nanoconfinement Effects on the Glass Transition and Crystallization Behaviors of Nifedipine. Mol. Pharmaceutics 2019, 16, 856–866. 10.1021/acs.molpharmaceut.8b01172. 30615456

[ref16] Wang F.; Hui H.; Barnes T. J.; Barnett C.; Prestidge C. A. Oxidized Mesoporous Silicon Microparticles for Improved Oral Delivery of Poorly Soluble Drugs. Mol. Pharmaceutics 2010, 7, 227–236. 10.1021/mp900221e. 19874003

[ref17] Cao Y.; Zhang K.; Wang M.; Gao Z.; Wang J.; Gong J. Influence of Adsorption State and Molecular Interaction on Physical Stability of Confined Amorphous Vortioxetine. Mol. Pharmaceutics 2021, 18, 2754–2763. 10.1021/acs.molpharmaceut.1c00288. 34152780

[ref18] Kramarczyk D.; Knapik-Kowalczuk J.; Smolka W.; Monteiro M. F.; Tajber L.; Paluch M. Inhibition of Celecoxib Crystallization by Mesoporous Silica–Molecular Dynamics Studies Leading to the Discovery of the Stabilization Origin. Eur. J. Pharm. Sci. 2022, 171, 106132 10.1016/j.ejps.2022.106132. 35077845

[ref19] Kawakami K. Nucleation and Crystallization of Celecoxib Glass: Impact of Experience of Low Temperature on Physical Stability. Thermochim. Acta 2019, 671, 43–47. 10.1016/j.tca.2018.11.004.

[ref20] Donth E. The Size of Cooperatively Rearranging Regions at the Glass Transition. J. Non-Cryst. Solids 1982, 53, 325–330. 10.1016/0022-3093(82)90089-8.

[ref21] Harada T.; Kawakami K.; Yoshihashi Y.; Yonemochi E.; Terada K.; Moriyama H. Practical Approach for Measuring Heat Capacity of Pharmaceutical Crystals/Glasses by Modulated-Temperature Differential Scanning Calorimetry. Chem. Pharm. Bull. 2013, 61, 315–319. 10.1248/cpb.c12-00928. 23449200

[ref22] Han X.; Dai K.; Kawakami K. Influence of Nucleation on Relaxation, Molecular Cooperativity, and Physical Stability of Celecoxib Glass. Mol. Pharmaceutics 2024, 21, 1794–1803. 10.1021/acs.molpharmaceut.3c01116. 38401048

[ref23] Knapik J.; Wojnarowska Z.; Grzybowska K.; Jurkiewicz K.; Stankiewicz A.; Paluch M. Stabilization of the Amorphous Ezetimibe Drug by Confining Its Dimension. Mol. Pharmaceutics 2016, 13, 1308–1316. 10.1021/acs.molpharmaceut.5b00903. 26981876

[ref24] Grzybowska K.; Capaccioli S.; Paluch M. Recent Developments in the Experimental Investigations of Relaxations in Pharmaceuticals by Dielectric Techniques at Ambient and Elevated Pressure. Adv. Drug Delivery Rev. 2016, 100, 158–182. 10.1016/j.addr.2015.12.008. 26705851

[ref25] Ahern R. J.; Hanrahan J. P.; Tobin J. M.; Ryan K. B.; Crean A. M. Comparison of Fenofibrate-Mesoporous Silica Drug-Loading Processes for Enhanced Drug Delivery. Eur. J. Pharm. Sci. 2013, 50, 400–409. 10.1016/j.ejps.2013.08.026. 23981335

[ref26] Malfait B.; Correia N. T.; Mussi A.; Paccou L.; Guinet Y.; Hédoux A. Solid-State Loading of Organic Molecular Materials within Mesoporous Silica Matrix: Application to Ibuprofen. Microporous Mesoporous Mater. 2019, 277, 203–207. 10.1016/j.micromeso.2018.10.022.

[ref27] Kaptay G. The Gibbs Equation versus the Kelvin and the Gibbs-Thomson Equations to Describe Nucleation and Equilibrium of Nano-Materials. J. Nanosci. Nanotechnol. 2012, 12, 2625–2633. 10.1166/jnn.2012.5774. 22755100

[ref28] Wunderlich B. Reversible Crystallization and the Rigid–Amorphous Phase in Semicrystalline Macromolecules. Prog. Polym. Sci. 2003, 28, 383–450. 10.1016/S0079-6700(02)00085-0.

[ref29] Bavnho̷j C. G.; Knopp M. M.; Madsen C. M.; Löbmann K. The Role Interplay between Mesoporous Silica Pore Volume and Surface Area and Their Effect on Drug Loading Capacity. Int. J. Pharm.: X 2019, 1, 100008 10.1016/j.ijpx.2019.100008. 31517273 PMC6733371

[ref30] Cordeiro T.; Matos I.; Danède F.; Sotomayor J. C.; Fonseca I. M.; Corvo M. C.; Dionísio M.; Viciosa M. T.; Affouard F.; Correia N. T. Evidence of Strong Guest–Host Interactions in Simvastatin Loaded in Mesoporous Silica MCM-41. Pharmaceutics 2023, 15, 1320 10.3390/pharmaceutics15051320. 37242562 PMC10222570

[ref31] Kawakami K.; Pikal M. J. Calorimetric Investigation of the Structural Relaxation of Amorphous Materials: Evaluating Validity of the Methodologies. J. Pharm. Sci. 2005, 94, 948–965. 10.1002/jps.20298. 15793805

[ref32] Cordeiro T.; Santos A. F. M.; Nunes G.; Cunha G.; Sotomayor J. C.; Fonseca I. M.; Danède F.; Dias C. J.; Cardoso M. M.; Correia N. T.; Viciosa M. T.; Dionísio M. Accessing the Physical State and Molecular Mobility of Naproxen Confined to Nanoporous Silica Matrixes. J. Phys. Chem. C 2016, 120, 14390–14401. 10.1021/acs.jpcc.6b04078.

[ref33] Azaïs T.; Tourné-Péteilh C.; Aussenac F.; Baccile N.; Coelho C.; Devoisselle J.-M.; Babonneau F. Solid-State NMR Study of Ibuprofen Confined in MCM-41 Material. Chem. Mater. 2006, 18, 6382–6390. 10.1021/cm061551c.

[ref34] Mishra S.; Gupta S. K.; Jha P. K.; Pratap A. Study of Dimension Dependent Diffusion Coefficient of Titanium Dioxide Nanoparticles. Mater. Chem. Phys. 2010, 123, 791–794. 10.1016/j.matchemphys.2010.05.061.

[ref35] Alcoutlabi M.; McKenna G. B. Effects of Confinement on Material Behaviour at the Nanometre Size Scale. J. Phys.:Condens. Matter 2005, 17, R461–R524. 10.1088/0953-8984/17/15/R01.

[ref36] Azais T.; Hartmeyer G.; Quignard S.; Laurent G.; Babonneau F. Solution State NMR Techniques Applied to Solid State Samples: Characterization of Benzoic Acid Confined in MCM-41. J. Phys. Chem. C 2010, 114, 8884–8891. 10.1021/jp910622m.

[ref37] Gignone A.; Delle Piane M.; Corno M.; Ugliengo P.; Onida B. Simulation and Experiment Reveal a Complex Scenario for the Adsorption of an Antifungal Drug in Ordered Mesoporous Silica. J. Phys. Chem. C 2015, 119, 13068–13079. 10.1021/acs.jpcc.5b02666.

[ref38] Wang K.; Sun C. C. Crystal Growth of Celecoxib from Amorphous State: Polymorphism, Growth Mechanism, and Kinetics. Cryst. Growth Des. 2019, 19, 3592–3600. 10.1021/acs.cgd.9b00597.

[ref39] Chawla G.; Gupta P.; Thilagavathi R.; Chakraborti A. K.; Bansal A. K. Characterization of Solid-State Forms of Celecoxib. Eur. J. Pharm. Sci. 2003, 20, 305–317. 10.1016/S0928-0987(03)00201-X. 14592696

[ref40] Ferro L. J.; Miyake P. S. Polymorphic Crystalline Forms of Celecoxib. US Patent 2004/0087640 A1, 2004.

[ref41] Kawakami K. Pharmaceutical Applications of Thermal Analysis. In Handbook of Thermal Analysis and Calorimetry “Recent Advances in Techniques and Applications”, 2nd ed.; Vyazovkin S.; Koga N.; Schick C., Eds.; Elsevier, 2018; pp 613–641. 10.1016/B978-0-444-64062-8.00009-7

[ref42] Martí J.; Sala J.; Guàrdia E. Molecular Dynamics Simulations of Water Confined in Graphene Nanochannels: From Ambient to Supercritical Environments. J. Mol. Liq. 2010, 153, 72–78. 10.1016/j.molliq.2009.09.015.

[ref43] Martí J.; Nagy G.; Guàrdia E.; Gordillo M. C. Molecular Dynamics Simulation of Liquid Water Confined inside Graphite Channels: Dielectric and Dynamical Properties. J. Phys. Chem. B 2006, 110, 23987–23994. 10.1021/jp0647277. 17125368

[ref44] Brás A. R.; Fonseca I. M.; Dionísio M.; Schönhals A.; Affouard F.; Correia N. T. Influence of Nanoscale Confinement on the Molecular Mobility of Ibuprofen. J. Phys. Chem. C 2014, 118, 13857–13868. 10.1021/jp500630m.

[ref45] Guenneau F.; Panesar K.; Nossov A.; Springuel-Huet M.-A.; Azaïs T.; Babonneau F.; Tourné-Péteilh C.; Devoisselle J.-M.; Gédéon A. Probing the Mobility of Ibuprofen Confined in MCM-41 Materials Using MAS-PFG NMR and Hyperpolarised-129Xe NMR Spectroscopy. Phys. Chem. Chem. Phys. 2013, 15, 18805–18808. 10.1039/c3cp52695j. 24100415

[ref46] Rams-Baron M.; Wojnarowska Z.; Grzybowska K.; Dulski M.; Knapik J.; Jurkiewicz K.; Smolka W.; Sawicki W.; Ratuszna A.; Paluch M. Toward a Better Understanding of the Physical Stability of Amorphous Anti-Inflammatory Agents: The Roles of Molecular Mobility and Molecular Interaction Patterns. Mol. Pharmaceutics 2015, 12, 3628–3638. 10.1021/acs.molpharmaceut.5b00351. 26323061

[ref47] Miranda D. F.; Zhang S.; Runt J. Controlling Crystal Microstructure to Minimize Loss in Polymer Dielectrics. Macromolecules 2017, 50, 8083–8096. 10.1021/acs.macromol.7b01450.

[ref48] Carpentier L.; Decressain R.; Desprez S.; Descamps M. Dynamics of the Amorphous and Crystalline α-, β-Phases of Indomethacin. J. Phys. Chem. B 2006, 110, 457–464. 10.1021/jp053545u. 16471556

[ref49] Kramarczyk D.; Knapik-Kowalczuk J.; Klimontko J.; Kurek M.; Jachowicz R.; Paluch M. Tuning the Physical State of Aripiprazole by Mesoporous Silica. Mol. Pharmaceutics 2024, 21, 2315–2326. 10.1021/acs.molpharmaceut.3c01095. PMC11080047 38644570

[ref50] Kołodziejczyk K.; Tarnacka M.; Kamińska E.; Dulski M.; Kamiński K.; Paluch M. Crystallization Kinetics under Confinement. Manipulation of the Crystalline Form of Salol by Varying Pore Diameter. Cryst. Growth Des. 2016, 16, 1218–1227. 10.1021/acs.cgd.5b01181.

[ref51] Talik A.; Tarnacka M.; Minecka A.; Hachula B.; Grelska J.; Jurkiewicz K.; Kaminski K.; Paluch M.; Kaminska E. Anormal Thermal History Effect on the Structural Dynamics of Probucol Infiltrated into Porous Alumina. J. Phys. Chem. C 2021, 125, 3901–3912. 10.1021/acs.jpcc.0c10560.

