# Supplementary material for: Influence of Pore Size of Mesoporous Silica on Physical Stability of Overloaded Celecoxib Glass
Source: Mol Pharm. 2025 Apr 4;22(5):2556–67. doi: 10.1021/acs.molpharmaceut.4c01482 (PMC12056689; doi:10.1021/acs.molpharmaceut.4c01482)
Supplement: Supplementary file 1 — mp4c01482_si_001.pdf [file mp4c01482_si_001.pdf]

SUPPORTING INFORMATION

Influence of Pore Size of Mesoporous Silica on Physical Stability of Overloaded Celecoxib Glass

Xue Han[1,2], Kohsaku Kawakami[1,2,*]

[1] Research Center for Macromolecules and Biomaterials, National Institute for Materials Science, 1-1 Namiki, Tsukuba, Ibaraki 305-0044, Japan

[2] Graduate School of Science and Technology, University of Tsukuba, 1-1-1 Tennodai, Tsukuba, Ibaraki 305-8577, Japan

* Corresponding author

E-mail: kawakami.kohsaku@nims.go.jp, Tel. +81-29-860-4424

Investigation on α relaxation and fragility

The T_g values of all the samples were determined by fitting the temperature dependency of the τ_α values to the VFT equation under the assumption of τ_α=100 s. The fragility (m) was calculated by the equation as follows[1]:

$$m = \left.\frac{d\log\tau_\alpha}{d\frac{T_g}{T}}\right|_{T=T_g} = \frac{D\left(\frac{T_0}{T_g}\right)}{\left(1-\left(\frac{T_0}{T_g}\right)\right)^2 \ln(10)} \qquad \text{(S1)}$$

Where, T_0 is the temperature analogous to the Kauzmann temperature, and D is Angell's strength parameter.

Table S1. Comparison of T_g, m, and VFT parameters of Pure CEL, Mixture with MS of different concentrations.

Sample	T_g (K) Onset of DSC	T_g (K) BDS	$\log\tau_\infty$	T_0 (K)	m
Pure CEL	328.0	328.6	−14.0	281.6 ± 0.8	111.6

Mixture with 25% SYL350	327.7	328.6	−14.0	282.4 ± 0.8	113.6
Mixture with 25% SYL730	328.2	328.5	−14.0	281.4 ± 0.4	111.5
Mixture with 67% SYL350	/	329.0	-14.0	282.3± 0.8	110.2
Mixture with 67% SYL730	/	329.3	-14.0	286.2± 1.1	122.2

As shown in Table S1, all the samples were fitted by an identical VFT function, the T_g values obtained by the VFT fitting agreed well with those determined by DSC, and the *m* of pure CEL was in good agreement with the reported results[2].

Reference

(1) Huang, D.; McKenna, G. B. New Insights into the Fragility Dilemma in Liquids. *J. Chem. Phys.* **2001**, *114*, 5621–5630.

(2) Grzybowska, K.; Paluch, M.; Grzybowski, A.; Wojnarowska, Z.; Hawelek, L.; Kolodziejczyk, K.; Ngai, K. L. Molecular Dynamics and Physical Stability of Amorphous Anti-Inflammatory Drug: Celecoxib. *J. Phys. Chem. B* **2010**, *114*, 12792–12801.
